# Concordance Study Between IBM Watson for Oncology and Real Clinical Practice for Cervical Cancer Patients in China: A Retrospective Analysis

**DOI:** 10.3389/fgene.2020.00200

**Published:** 2020-03-24

**Authors:** Fang-wen Zou, Yi-fang Tang, Chao-yuan Liu, Jin-an Ma, Chun-hong Hu

**Affiliations:** ^1^Department of Oncology, The Second Xiangya Hospital of Central South University, Changsha, China; ^2^Department of Anesthesiology, The Second Xiangya Hospital of Central South University, Changsha, China

**Keywords:** artificial intelligence, Watson for Oncology, cervical cancer, concordance, chian

## Abstract

Watson for Oncology (WFO) is a artificial intelligence clinical decision-support system with evidence-based treatment options for oncologists. WFO has been gradually used in China, but limited reports on whether WFO is suitable for Chinese patients. This study aims to investigate the concordance of treatment options between WFO and real clinical practice for Cervical cancer patients retrospectively. We retrospectively enrolled 300 cases of cervical cancer patients. WFO provides treatment options for 246 supported cases. Real clinical practice were defined as concordant if treatment options were designated “recommended” or “for consideration” by WFO. Concordance of treatment option between WFO and real clinical practice was analyzed statistically. The treatment concordance between WFO and real clinical practice occurred in 72.8% (179/246) of cervical cancer cases. Logistic regression analysis showed that rural registration residences, advanced age, poor ECOG performance status, stages II-IV disease have a remarkable impact on consistency. The main reasons attributed to the 27.2% (67/246) of the discordant cases were the substitution of nedaplatin for cisplatin, reimbursement plan of bevacizumab, surgical preference, and absence of neoadjuvant/adjuvant chemotherapy and PD-1/PD-L1 antibodies recommendations. WFO recommendations were in 72.8% of concordant with real clinical practice for cervical cancer patients in China. However, several localization and individual factors limit its wider application. So, WFO could be an essential tool but it cannot currently replace oncologists. To be rapidly and fully apply to cervical cancer patients in China, accelerate localization and improvement were needed for WFO.

## Introduction

Artificial intelligence (AI) is the frontier and dominating terrain of Information Technology which able to simulate human mental status and cognitive function (Jiang et al., 2017). With the development of AI and medical diagnosis technology, clinical decision-support systems (CDSS) with intelligent diagnostic function has become one of the important issues of science for medical information (Meyer et al., 2018). Watson for Oncology (IBM) is a representative AI CDSS that developed by IBM Co.Ltd in United States. WFO can provide a reasonable individualized treatment plan for cancar patients by obtaining valuable information from medical records. WFO first officially landed in China in 2016, until now, more than 80 hospitals use WFO as an important medical diagnostic tool for individualized treatment of tumor (IBM, 2017). WFO can provide counseling services for almost all cancer patients. However, whether WFO was fit for Chinese cancer patients, especially cervical cancer patients.

Cervical cancer is common in the female genital tract malignant tumors, and the incidence of which is second only to that of breast cancer among women worldwide, making it the second-most serious cancer threatening the health and lives of women (Jassim et al., 2018). Compared to breast cancer, cervical cancer is more common in developing countries due to poor health status, and it is the most common in China (Gu X. Y. et al., 2018). And rural and remote areas are also a prevalent regions for cervical cancer in China. But the current problem of the medical service is that the main hospitals hold too many premium resources, but in the meantime, the primary health agencies are excessively lack of resources (Bao et al., 2018). Cervical cancer patients in rural and remote areas can not reach the effective treatment recommendation, especially at centers where cancer expert resources are limited. So, WFO is of great significance for Chinese patients with cervical cancer, especially patients in rural and remote areas with limited medical resources.

Therefore, we conducted a retrospective and observational study on cervical cancer at The Second Xiangya Hospital Cancer Center to explore consistency between WFO and clinical treatment recommendations supported by an expert panel of cancer specialists for Cervical cancer patients.

## Materials and Methods

### Study Population

**FIGURE 1:**
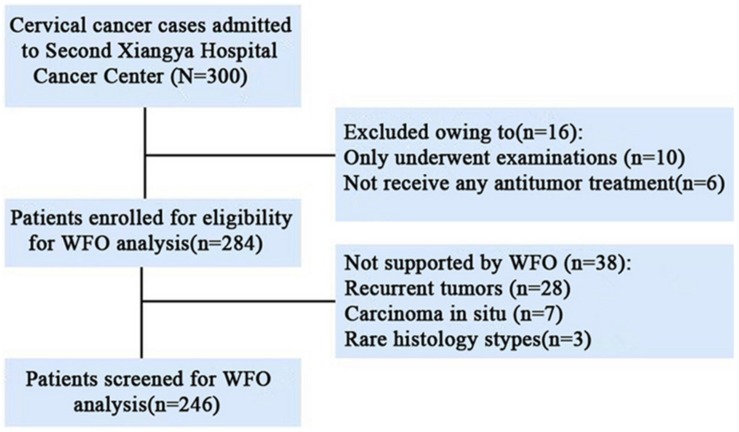
CONSORT diagram. WFO, Watson for Oncology.

This retrospective study was reviewed and approved by the Medical Ethics Committee of The Second Xiangya Hospital of Central south university (approval number was 2017-S104). We retrospectively and randomly selected 300 cases of cervical cancer patients from 05/2016 to 08/2018. All patients with cervical cancer confirmed by pathology at The Second Xiangya Hospital Cancer Center. Untreated Patients and recurrent tumors, rare histology that not yet trained to offer treatment options by WFO system were excluded. A total of 18% (54/300) cases excluded from our study and 82% (246/300) cases were included in our study. The detailed patient selection process is shown in Figure 1.

### Watson for Oncology

Watson for Oncology (IBM Corporation, United States, version 18.1R) used in our study were provided by Baheal Intelligent Technology Co., Ltd^1^. The clinicopathologic data of supported cases were extracted from medical records and entered into the WFO system. Treatment options recommended by WFO were presented in three categories: Blue represents “Recommended” with a strong evidence supported, Orange represents “For consideration” with a potentially suitable evidence-based alternative considered by oncologists based on their clinical judgment, and Red represents that is “Not recommended” that a treatment with contraindications or strong evidence against its use.

### Real Clinical Practice for Cervical Cancer

The Second Xiangya Hospital Cancer Center one of the biggest and best oncology departments in the Hunan Province of China. Gynecological Oncology Center is the most important part of The Second Xiangya Hospital Cancer Center and mainly serves cervical cancer, ovarian cancer, endometrial cancer, and other gynecological malignant tumors. Gynecological Oncology Center has a multidisciplinary team (MDT) composed of oncologists, gynecologists, radiologists, pathologists, and nutritionists, et al. MDT forms and implements a comprehensive regimen based on NCCN guidelines and the patient’s specific conditions. This comprehensive regimen was considered to be a real clinical practice for cervical cancer.

### Data Acquisition and Concordance Judgment

**FIGURE 2:**
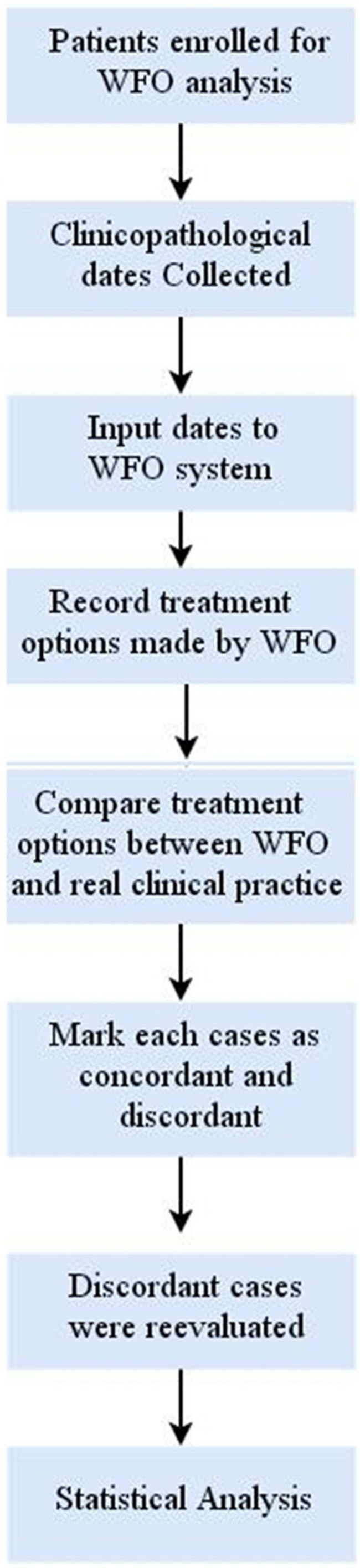
Flow diagram of the study design. WFO, Watson for Oncology.

The available clinicopathologic data of 246 patients included a registered residence, age, performance status, pathological type, differentiation degree, FIGO stage, lymphatic and distant metastasis, HPV status, and detailed clinical treatment plan were collected from Second Xiangya Hospital Cancer Center clinical electronic medical records and inputted into WFO system by 2 oncologists manually. Treatment options generated by WFO and recorded through two trained oncologists. It should be noted that in the data analysis process, real clinical practice were categorized as concordant if treatment options were designated “recommended” or “for consideration” by WFO. And if the real clinical practice was not recommended by WFO or if WFO did not provide the same treatment options, the recommendations were considered as discordant. The discordant cases were reevaluated by two senior oncologists provided their reasons for choosing the real treatment options. The specific study design and procedures and are shown in Figure 2.

### Statistical Analysis

SPSS20.0 statistics software (SPSS, United States) and Microsoft Excel (2012) were employed to undergo statistical analysis. Descriptive statistics of 246 patients were calculated and presented as means ± standard (x ± s) or median. Differences between the clinicopathological characteristics of the groups were analyzed by Pearson’s χ^2^ test. Correlation between real clinical practice and WFO recommendations were assessed by the chi-square test. A logistic regression model was estimated with odds ratios (OR) and 95% confidence intervals (CIs). Thep values were designated as ^∗^*P* < 0.05.

## Results

### Clinicopathological Characteristics of Supported Cases

Of the 300 accrued cervical cancer patients, 246 patients were eligible for WFO analysis. Overall, 82% (246/300) of our enrolled cases were supported by WFO. Clinicopathological characteristics of 246 supported cases are detailed in Table 1. Among the 246 supported cases in our study, median age was 53 years (range, 35–78 years), and rural registration patients, stage II/II disease, squamous cell carcinoma, middle/poorly differentiated accounted for 66.2% (165/246), 77.7% (101 + 90/246), 89.0% (219/246), and 80.6% (90 + 108/246), respectively.

**TABLE 1:** Clinicopathological characteristics of cervical cancer patients (*N* = 246).

Clinicopathological characteristics	Total cases	Concordant cases
**Age, years, n (%)**		
≤45	29 (11.8)	25 (86.2)
45–65	165 (67.1)	134 (81.2)
≥65	52 (21.1)	20 (38.5)
Median age (range)	53(35−78)	–
**Registered residence, n (%)**		
Urban registration	81 (33.8)	78 (96.3)
Rural registration	165 (66.2)	101 (61.2)
**ECOG^a^ performance status, n (%)**		
0–1 points	186 (75.6)	145 (77.9)
2 points	47 (19.1)	31 (66.1)
≥3 points	13 (5.3)	3 (23.1)
**FIGO stage, n (%)**		
I	29 (11.8)	12 (41.4)
II	101 (41.1)	87 (86.1)
III	90 (36.6)	78 (86.7)
IV	26 (10.5)	2 (7.96)
**Lymphatic metastasis, n (%)**		
Positive	114 (46.3)	82 (71.9)
Negative	132 (53.7)	97 (73.5)
**Distant metastasis, n (%)**		
Positive	19 (7.7)	10 (52.6)
Negative	227 (92.3)	169 (74.5)
**Pathological types, n (%)**		
Squamous cell carcinoma	219 (89.0)	159 (72.6)
Adenocarcinoma	16 (6.5)	125 (75)
Adenosscale squamous cell carcinoma	10 (4.1)	7 (70)
Small cell carcinoma	1 (0.4)	1 (100)
**Differentiation degrees, n (%)**		
High differentiation	48 (19.5)	35 (72.9)
Middle differentiation	90 (36.6)	64 (71.1)
Poorly differentiation	108 (43.9)	80 (74.1)

*^*a*^Eastern Cooperative Oncology Group, ECOG.*

### Concordance Between WFO and Real Clinical Practice

After reevaluated by two senior oncologists of discordant cases, there was no change to the primary concordance. Overall treatment concordance between WFO and real clinical practice occurred in 72.8% (179/246) of cervical cancer cases, among the concordant cases, treatment options that designated “Recommended” or “For consideration” by WFO accounted for 41.5% (102/246) and 31.3% (77/246), respectively. Also, there were 27.2% (67/246) of case cannot consistent with real clinical practice, among the discordant cases, treatment options that not recommended by WFO or did not provided by WFO accounted for 4.8% (12/246) and 22.4% (55/246), respectively. Dates are shown in Table 2.

**TABLE 2:** Concordance between WFO and real clinical practice (*N* = 246).

Supported cases	Recommendations	Availability	Total
Concordant cases, n (%)	102 (41.5)^a^	77 (31.3)^b^	179 (72.8)
Discordant cases, n (%)	12 (4.8)^c^	55 (22.4)^d^	67 (27.2)

*^*a*^Recommended. ^*b*^For consideration. ^*c*^Not recommended. ^*d*^Not available.*

### Subgroup Analyses

**FIGURE 3:**
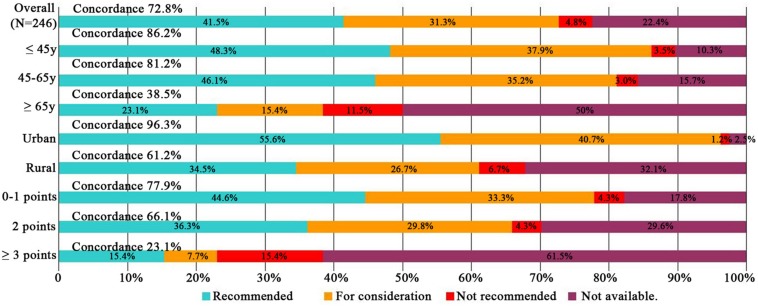
Treatment concordance between WFO and real clinical practice, divided by age, registered residence, and ECOG performance status. WFO, Watson for Oncology.

**FIGURE 4:**
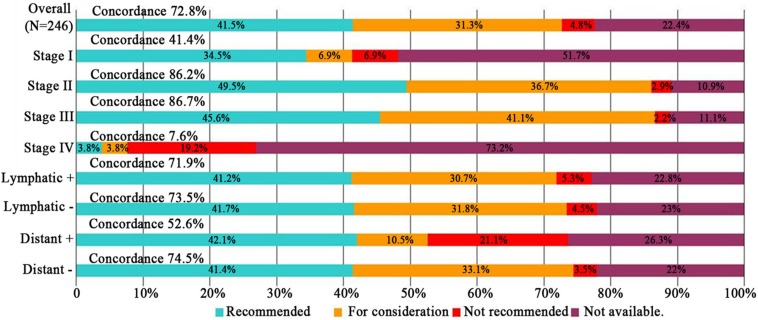
Treatment concordance between WFO and real clinical practice, divided by FIGO stage, lymphatic and distant metastasis. WFO, Watson for Oncology.

**FIGURE 5:**
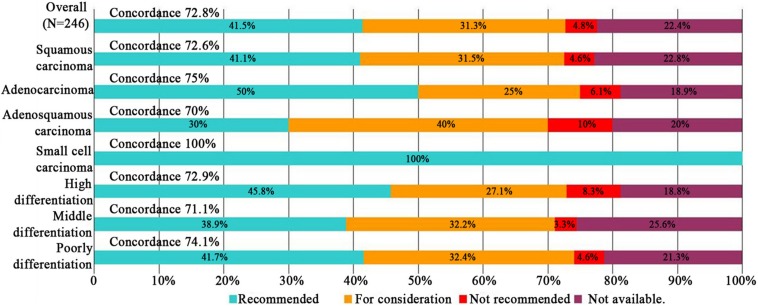
Treatment concordance between WFO and real clinical practice, divided by pathological types and differentiation degrees.

Subgroup analyses of treatment concordance with clinicopathological characteristics were also carried out. The result showed that urban registration patients [96.3% (78/84)], low age group (≤45 years and 45–65 years groups) [86.2% (25/29), 81.2% (134/165), respectively], good ECOG performance status (0–1 and 2 points groups) [77.9% (145/186), 66.1% (31/47), respectively], and stage II/III disease [80.2% (87/101), 86.7% (78/90), respectively] exhibiting higher concordance than rural registration patients [61.2% (101/165)], advanced age group (≥60 years) [38.5% (20/52)], poor ECOG performance status (≥3 points) [23.1% (3/13)], and stage I/IV disease[41.4% (12/29), 7.96% (2/26), respectively]. While, there were no obvious difference among lymphatic and distant metastasis disease, pathological types, differentiation degrees. Dates shown in Figures 3–5.

### Logistic Regression Analysis

The logistic regression analysis showed that, compared with patients ≤45 years of age, concordance declined significantly in patients ≥65 years of age and older[0.08 (0.03–0.28), *P* = *0.032*]. And Concordance was particularly low for patients with rural registration[0.64 (0.427–0.946), *P* = *0.025*],compare with urban patients. Poor ECOG performance status (≥3 points) patients exhibiting lower concordance than good ECOG performance status patients[0.29 (0.083–1.058), *P* = *0.048*]. Odds ratios of concordance varied by stage, showed that compared with stage I disease, stages II-III disease were significantly more likely to be concordant ([2.08 (1.002–4.325), *P* = *0.046*],[2.09 (1.001–4.381), *P* = *0.047*], respectively), whereas, concordance declined remarkably in stages IV disease [0.19 (0.038–0.91), *P* = *0.025*]. While, lymphatic and distant metastasis disease, pathological types, differentiation degrees were not found to affect concordance. Dates are shown in Table 3.

**TABLE 3:** Logistic regression model of concordance between Watson for Oncology and real clinical practice (*N* = 246).

Clinicopathological characteristics	OR^b^ (95%CIs^c^)	χ^2^	*P* value
Registered residence (Urban and Rural)	0.64 (0.427–0.946)	5.017	0.025*
Lymphatic metastasis (P^d^ and N^e^)	1.02 (0.694–1.503)	0.012	*0.913*
Distant metastasis (P^d^ and N^e^)	1.41 (0.641–3.12)	0.744	*0.388*
**Age, years**			
≤45 (Reference)	1.00	–	*–*
45–65	0.94 (0.527–1.685)	0.041	*0.841*
≥65	0.08 (0.03–0.28)	4.609	0.032*
**ECOG^a^ performance status**			
0–1 points (**Reference**)	1.00	–	–
2 points	0.84 (0.512–1.399)	0.425	*0.514*
≥3 points	0.29 (0.083–1.058)	3.917	*0.048*
**FIGO stage**			
I (**Reference**)	1.00	–	–
II	2.08 (1.002–4.325)	3.968	0.046*
III	2.09 (1.001–4.381)	3.958	0.047*
IV	0.19 (0.038–0.91)	5.036	0.025*
**Pathological types**			
Squamous cell carcinoma (**Reference**)	1.00	–	–
Adenocarcinoma	1.03 (0.476–2.244)	0.007	*0.935*
Adenosscale squamous cell carcinoma	0.96 (0.359–2.588)	0.005	*0.942*
Small cell carcinoma	1.38 (0.086–22.187)	0.051	*0.821*
**Differentiation degrees**			
High differentiation (**Reference**)	1.00	–	–
Middle differentiation	0.97 (0.568–1.675)	0.008	*0.928*
Poorly differentiation	1.01 (0.602–1.714)	0.003	*0.953*

^*a*^Eastern Cooperative Oncology Group, ECOG. ^*b*^Odds ratio, OR. ^*c*^Confidence intervals, CIs. ^*d*^Positive and^*e*^Negative. * *P* < 0.05.

### Analysis of Reasons for Discordant Cases

There were four critical factors attributed to 27.2% (67/246) of the discordant cases. Firstly, Cisplatin is the main chemotherapy drug recommended by WFO, but in our study, of 46.4% (31/67) cases select nedaplatin due to cannot tolerate gastrointestinal reactions of cisplatin. Next, bevacizumab as a routine option recommended by WFO for stage IV stage, but bevacizumab is not in medical reimbursement plan for cervical cancer in China, of 26.9% (18/67) patients reject bevacizumab therapy for the financial burden. Thirdly, for stage Ib2 and IIb disease, only concomitant radiochemotherapy was recommended by WFO, in our study, of 19.4% (13/67) patients prefer surgical therapy instead of concomitant radiochemotherapy. Moreover, neoadjuvant/adjuvant chemotherapy and programmed death-1 and ligand antibodies (PD-1/PD-L1 antibodies) drugs recommendations are not included in the WFO system, in our study, there were 9.1% (6/67),2.8% (2/67) patients chose neoadjuvant/adjuvant chemotherapy and pembrolizumab therapy. Dates are shown in Table 4.

**TABLE 4:** Analysis of reasons for discordant cases (*N* = 67).

Reasons for discordant cases	Cases, n (%)
Substitution of nedaplatin for cisplatin	28 (41.8)
Reimbursement plan of bevacizumab	18 (26.9)
Surgical preference	13 (19.4)
Neoadjuvant/adjuvant chemotherapy	6 (9.1)
PD-1/PD-L1 antibodies	2 (2.8)

## Discussion

From 2013, concordance studies between WFO and physicians have been performed in various countries and cancer types. A double-blind study showed that 93% concordance rate for 638 breast cancer patients (Kaur and Singh Mann, 2018; Somashekhar et al., 2018). A retrospective study from India for 1000 consecutive cases showed 80% concordance between multidisciplinary team (MDT) (Baek et al., 2017). A observational study from Korea showed a 73% concordance rate for colon cancer and a 49% concordance rate for gastric cancer (Somashekhar et al., 2016; Suwanvecho et al., 2017). And, a comparative Study from Korea indicated that WFO without the gene expression assay has limited clinical utility (Kim et al., 2018). It appears that the concordance results varies by countries and cancer types (Zhou et al., 2018). For China, a huge population and regional differences created a different therapeutic experiences and considerations for cancer patients, as well as large differences with Western countries. Also, a retrospective study (Liu et al., 2018) reported by our center revealed that treatment concordance between WFO and MDT occurred in 65.8% (98/149) of lung cancer. Another retrospective study (Zhou et al., 2019) from China showed that Ovarian cancer, lung cancer and breast cancer obtained a high concordance, the concordance of gastric cancer was very low, Incidence and pharmaceuticals may be the major cause of discordance. However, limited reports on whether WFO is suitable for Chinese cervical cancer patients, Zhou et al. reported 14 cervical cancer patients in this study, but the sample size is too small.

Our retrospective study provides the first evidence that accelerates localization and improvement were needed for WFO before comprehensive application in cervical cancer patients in China. Although treatment options generated by WFO were mostly concordant with real clinical practice, there are still unresolved issues. Firstly, as mentioned in the manual (Gu X. et al., 2018), some clinical settings are not yet supported by WFO system. In our study, of 73.7% (28/38) unsupported cases were recurrent tumors patients. But compare with our center, grass-roots hospitals have a greater proportion of patients with recurrent tumors. So, the cases that cannot be supported by WFO system are very large for cervical cancer patients in China. Secondly, localization factors such as physical of patients, medical reimbursement plan, economic condition, and patient preferences of China were different from western countries, and they ultimately affect the inconsistency. In our study, of 46.4% (31/67) cases select nedaplatin due to cannot tolerate gastrointestinal reactions of cisplatin, of 26.9% (18/67) patients reject bevacizumab therapy for financial burden. of 19.4% (13/67) patients prefer surgical therapy instead of concomitant radiochemotherapy. Moreover, registered residence, age, performance status, FIGO stage have a remarkable impact on consistency. Urban registration patients, low age group, good performance status, and stage II/III disease exhibiting higher concordance than rural registration patients, advanced age group, poor performance status, and stage I/IV disease. These personal factors make WFO unable to achieve individualized treatment and affect the consistency significantly in China. Finally, neoadjuvant/adjuvant chemotherapy (Sun et al., 2018). Chemotherapeutic drugs Goffin et al. (2010) such as gemcitabine, docetaxel, mitomycin, irinotecan, pemetrexed, vinorelbine, and PD-1/PD-L1 antibodies (Kim et al., 2017) drugs recommendations that performed in real clinical practice are not included in the WFO system.

Compared with previous research, our study provides the first evidence that WFO is not suitable for Chinese cervical cancer patients currently, and the sample size of this study was the largest among all cervical cancer studies performed. Also, we not only reported the consistency between WFO and real clinical practice, but also analyzed several influence elements and offered certainly advises for the improvement of WFO to better suit Chinese patients. But, our study contains some limitations. Firstly, this was a retrospective and observational study with control groups lacked, several unmeasured elements may influence the outcome. Secondly, treatment preferences among different experts also affect consistency. Thirdly, the distribution of clinicopathological characteristics among patients is imbalanced, for example, fewer patients were stage IV diseases may lead to a large disagreement for Stage IV tumors. Finally, molecular parameters, such as mutations, gene expression or protein localization can affect the treatment decision. But, in China, unlike lung cancer and breast cancer, gene detection were lacked for cervical cancer. Although there are some targeted drugs that may be effective for cervical cancer, such as PARP inhibitors (for BRCA1 or BRCA2 mutations patients), EGFR tyrosine kinase inhibitors (for EGFR mutations patients), gene detection is still not widely used in China. So, in our study, Because of the lack of gene detection datas, we cannot observe the effect of molecular parameters on treatment decisions.

For WFO, WFO could be an essential tool for clinicians, provides good references and literature for medical students, or even give some treatment advice to non-specialist (Malin, 2013; Werner et al., 2016). However, we believe that human physicians will not be replaced by AI in the foreseeable future, WFO still has a long way to go to replace oncologists. Medicine is not just a science, but also a social and psychological subject. Any tool and guidelines can only be used as a doctor’s reference, localization factors and individual elements should considered for different patients, especially for cancer patients with large heterogeneous (Kemin et al., 2017). Therefore, WFO must be significantly improved to adapt the real clinical practice in different countries. Patient’s physical and mental state, economic situation, complications, patient’s treatment preference and medical reimbursement plan in different countries should be taken into account and not just provide advice based on existing knowledge. For China, a unique medical database with Chinese characteristics should be created by WFO to adapt and serve Chinese cancer patients.

## Conclusion

In conclusion, WFO recommendations were in 72.8% of concordant with real clinical practice for cervical cancer patients in China. However, several localization and individual factors limit its wider application. So, WFO cannot replace oncologists for cervical cancer patients in China currently. WFO could be an effective decision-support tool in cancer therapy for Chinese physicians, it also helps to standardize the treatment of cervical cancer. To be rapidly and fully apply to cervical cancer patients in China, accelerate localization and improvement were needed for WFO.

## Data Availability Statement

The datasets generated for this study are available on request to the corresponding author.

## Ethics Statement

This retrospective study was reviewed and approved by the Medical Ethics Committee of The Second Xiangya Hospital of Central South University (approval number was 2017-S104).

## Author Contributions

CH was responsible for overall planning for research. FZ was responsible for data collection and statistical analysis. YT and CL were involved in data analysis. JM was participated in the preparation of manuscript.

## Conflict of Interest

The authors declare that the research was conducted in the absence of any commercial or financial relationships that could be construed as a potential conflict of interest.

## Footnotes

1 https://www.bsmartd.com
